# The Sub-Annual Breeding Cycle of a Tropical Seabird

**DOI:** 10.1371/journal.pone.0093582

**Published:** 2014-04-08

**Authors:** S. James Reynolds, Graham R. Martin, Alistair Dawson, Colin P. Wearn, B. John Hughes

**Affiliations:** 1 Centre for Ornithology, School of Biosciences, College of Life and Environmental Sciences, University of Birmingham, Edgbaston, Birmingham, United Kingdom; 2 Army Ornithological Society, Land Forces Directorate, Army Headquarters, Andover, Hampshire, United Kingdom; 3 Centre for Ecology and Hydrology, Penicuik, Midlothian, United Kingdom; 4 Royal Air Force Ornithological Society, Headquarters Air Command, Royal Air Force, High Wycombe, Buckinghamshire, United Kingdom; Behavioural Ecology & Ecophysiology group, Denmark

## Abstract

Breeding periodicity allows organisms to synchronise breeding attempts with the most favourable ecological conditions under which to raise offspring. For most animal species, ecological conditions vary seasonally and usually impose an annual breeding schedule on their populations; sub-annual breeding schedules will be rare. We use a 16-year dataset of breeding attempts by a tropical seabird, the sooty tern (*Onychoprion fuscatus*), on Ascension Island to provide new insights about this classical example of a population of sub-annually breeding birds that was first documented in studies 60 years previously on the same island. We confirm that the breeding interval of this population has remained consistently sub-annual. By ringing >17000 birds and re-capturing a large sample of them at equivalent breeding stages in subsequent seasons, we reveal for the first time that many individual birds also consistently breed sub-annually (i.e. that sub-annual breeding is an individual as well as a population breeding strategy). Ascension Island sooty terns appear to reduce their courtship phase markedly compared with conspecifics breeding elsewhere. Our results provide rare insights into the ecological and physiological drivers of breeding periodicity, indicating that reduction of the annual cycle to just two life-history stages, breeding and moult, is a viable life-history strategy and that moult may determine the minimum time between breeding attempts.

## Introduction

The periodicity of animal breeding has long fascinated biologists who have often invoked ecological explanations of within and between-species variation in the timing of breeding and its evolution (e.g. [1], [2]). Timing of breeding and its periodicity are often viewed as adaptations that promote the maximum survival of offspring through the synchronisation of a breeding attempt with ecologically favourable conditions which usually result in high food availability [3], [4]. Most parts of the world are ‘seasonal’ with regard to key factors that determine biological productivity (mainly temperature, precipitation and day length) and that result in peaks of food availability within an annual cycle. In most situations there is only one period of peak food availability in the year and thus the breeding patterns of most animal species that live longer than one year will be annual.

Of all life-history stages, reproduction is probably most sensitive to an organism's ecology. This is true for almost all animal taxa that combat unpredictability of resources by interrupting breeding at different reproductive thresholds (e.g. some insects) and delaying implantation of embryos (e.g. some mammals). However, such plasticity in the timing of breeding is not exhibited by birds [5], many of which undergo extensive physiological and behavioural preparations for breeding with its timing modulated by proximate factors such as temperature and photoperiod [4], [6]. There is extensive knowledge of the ecological and physiological factors associated with breeding periodicity in birds which breed annually at temperate latitudes. However, such knowledge of non-annual breeders is relatively poor. In some seabirds, such as great albatrosses (*Diomedea* spp.) and frigatebirds (*Fregata* spp.), there is annual breeding at colonies but it is well established that individuals within those populations breed only biennially [7], [8] which is explained by the extended breeding period of individuals which have to wait for the predictable occurrence of resources in a second season after they have bred successfully [9]. Other seabirds on tropical islands breed sub-annually. These include species whose populations breed every six months such as swift terns (*Sterna bergii*) on Aldabra Atoll [10] and white terns (*Gygis alba*) on Christmas Island (Pacific) [11], every seven to eight months such as white-tailed tropicbirds (*Phaethon lepturus*) on Ascension Island [12] and Aldabra Atoll [13], and bridled terns (*Sterna anaethetus*) on Aldabra Atoll [10], and every nine to 10 months such as Christmas shearwaters (*Puffinus nativitatis*) on the Pitcairn Islands [14], and Audubon's shearwaters (*Puffinus lherminieri*) and swallow-tailed gulls (*Creagrus furcatus*) on The Galapagos Islands [15], [16].

All birds undergo cycles involving both breeding and moult which together impose some temporal constraints on the timing of breeding [4]. In addition, food availability also constrains the timing of breeding and even in the tropics seasonal changes in abundance and distribution of food may regulate breeding cycles in some seabirds [17]. Avian breeding seasons are often longer in tropical than temperate zones [18] because productivity is lower, food more widely distributed and peaks in its abundance are less well defined temporally [19]. Some tropical seabird populations contain individuals which breed at any time of the year (e.g. red-footed boobies [*Sula sula*], white terns and red-billed tropicbirds [*Phaethon aethereus*] on Johnston Atoll in the central Pacific [17]; red-billed and white-tailed tropicbirds on Ascension Island [12]).

The breeding interval of populations is not always the same as that of constituent individuals. For example, while individual white terns [11], Christmas shearwaters [14], Audubon's shearwaters [15] and swallow-tailed gulls [16] have been observed breeding sub-annually, their populations breed year-round. Furthermore, there is considerable geographic variation in the breeding interval of some species. For example, individual white-tailed tropicbirds consistently breed sub-annually throughout their range except in Bermuda where they breed annually [13]. While some studies have described in detail the sub-annual breeding interval of tropical seabird populations, they tend to be only over a few years and there is a lack of robust longitudinal data on the breeding intervals of individual birds in relation to that of their populations. For example, on the British Ornithologists' Union's (BOU's) Centenary Expedition to Ascension Island in the South Atlantic between 1957 and 1959 Stonehouse [12] ringed 1400 tropicbirds and showed that their breeding intervals varied between five and 10 months in white-tailed tropicbirds and between nine and 12 months in red-billed tropicbirds, depending upon the breeding outcome. Successful breeders had longer breeding intervals but the investigation was limited to the 18 month duration of the expedition and so more sustained patterns of breeding could not be determined.

The classical example of sub-annual breeding at a population level is provided by the work of Chapin [20] in the 1950s who found that the average breeding interval of the Ascension Island population of sooty terns was 292.6 days (or 9.61 months) with the initiation of egg laying across the whole population lasting approximately 10 weeks. In response to these findings Lack [1] noted: “Elsewhere, so far as known, the breeding season of this widespread species is not unusual, but on Ascension Island the birds return for the ‘Wideawake Fair’ at an average interval of just over 9 months… The factors involved are not known.” Most sooty tern populations breed annually [21], but rarely they have been recorded as breeding with six month (Christmas Island [22]) or 10 month (e.g. Australia [23]) intervals. Ashmole [24] significantly advanced understanding of sub-annual breeding of sooty terns on Ascension Island by ringing birds and showing that some laid in successive breeding seasons and completed moult in between. However, he ringed only several hundred birds in one season and re-captured only 63 of them in the following season. Like the findings of Stonehouse [12], his suffered from the brevity of the BOU expedition and from a strong El Niño-Southern Oscillation (ENSO) event in 1957–1958 (see table 7.1 in Schreiber [25]) that may have reduced food availability prior to and during the BOU expedition. However, how the ENSO influences the reproductive biology of seabirds remains poorly understood. Together, these features of the 1957–1959 expedition to Ascension Island cast doubt on sub-annual breeding being a regular feature of the breeding biology of populations, and especially individuals, of this species.

Some such as Newton [26] have strongly argued that there is a need for more reliable data at both individual and population levels before sub-annual breeding can be discussed more critically as a breeding strategy of birds. Here, we test three hypotheses concerning the breeding interval of sooty terns on Ascension Island. We use a 16-year dataset of first hatching dates and a ringing - re-capture dataset of >17000 birds and examine the breeding interval at population and at individual levels. We hypothesised that the breeding interval of the population (1) and of most individuals in the population (2) is consistently less than annual. Furthermore, we hypothesised that some or all of the constituent phases of the breeding cycle of sooty terns breeding on Ascension Island differ markedly in length than those of annually breeding conspecifics which breed annually elsewhere (3). By comparing the breeding cycle of sooty terns on Ascension Island with that of conspecifics elsewhere, we progress understanding of how some bird species and populations may consistently breed sub-annually and we discuss how this sub-annual breeding cycle may be maintained.

## Materials and Methods

### Study Area and Species

The study was carried out on Ascension Island (07°57′S, 14°24′W), a 97 km^2^ volcanic island and a constituent of the United Kingdom Overseas Territory (UKOT) of St Helena. It is isolated in the tropical South Atlantic midway between West Africa and South America. Although many scientific expeditions have visited the island, including The Beagle in 1838 [27], few have remained sufficiently long to provide thorough accounts of the breeding biology of avifauna. Nevertheless, historical records suggest that seabirds once bred in large numbers on Ascension Island (e.g. [28]) with the island providing the only breeding location in over 2.6 million km^2^ of ocean. Sooty terns now constitute over 95% of avian biomass of the island with possibly as many as 750000 birds breeding in the 1950s [24]. Since 1990 the population has varied between 138000±5000 (95% confidence limits) and 420000±7000 birds [29]. Sooty terns are seabirds of tropical oceans that nest in large dense colonies on remote oceanic islands throughout the equatorial zone [21].

### Field Methods

We monitored the interval between first hatching dates across 19 breeding cycles between 1996 and 2012 (inclusive) to establish the breeding interval of the populations at Mars Bay and Waterside colonies on the southern coast of the island (see figure 1 in Hughes et al. [30]). Each colony comprises many sub-colonies within which breeding synchrony is strong but between which it is much weaker [30]. This is typical for many seabird species nesting in large colonies [31]. Each season the first sub-colony to be occupied was identified by observing pre-laying behaviour of all returning birds at both colonies. Observations of birds were made by the Army Ornithological Society (AOS) that has been mounting expeditions to the island since 1990 and they were supplemented with information from staff at the island's Meteorological and Conservation Offices, and from personal communications with contract workers. Individual nests were not monitored for first hatching dates but all sub-colonies were surveyed for stage of breeding (i.e. pre-laying, incubation, brooding and feeding chicks). Observations of egg desertion, indicating freshly laid eggs, and of embryo age from freshly predated broken eggs allowed determination of egg age within each sub-colony.

**Figure 1:**
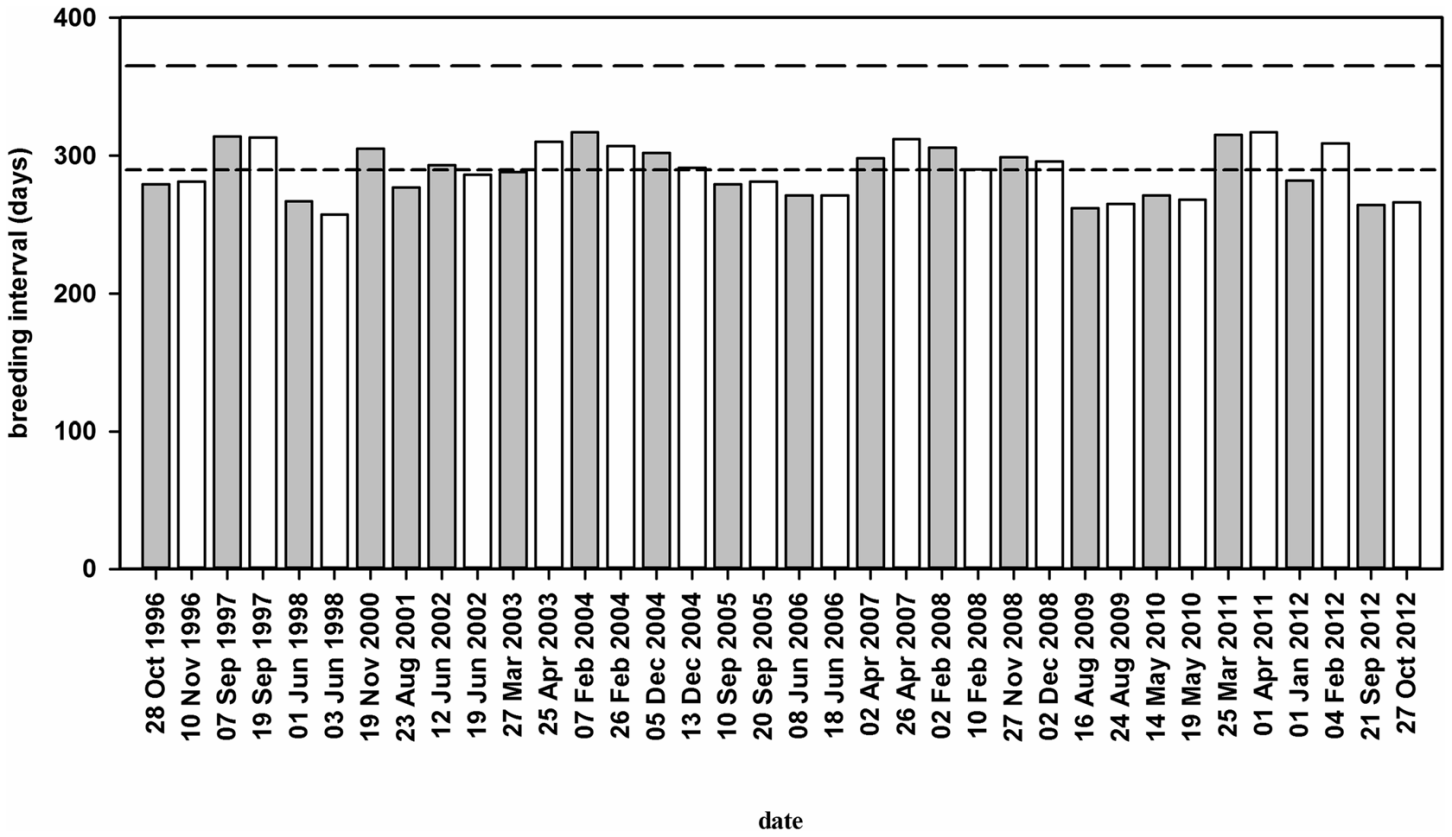
Breeding intervals of the population of sooty terns on Ascension Island. Breeding intervals plotted against date of first hatching of the sooty tern populations breeding at Mars Bay (open bars) and Waterside (grey bars) colonies on Ascension Island in the South Atlantic. Short-dashed line indicates a sub-annual breeding interval of 9.6 months while long-dashed line indicates one that is annual.

We captured 17426 incubating adults between 1996 and 2012 (inclusive) using long-handled nets and ringed them (under a British Trust for Ornithology [BTO] ringing licence number A4318) with uniquely numbered BTO metal rings to study the breeding interval of individual birds. Birds were generally ringed in groups of 100 within a sub-colony before moving on to another sub-colony. This was done to minimise disruption to incubating birds. In a given season re-capture effort took place before ringing of birds to avoid re-capture of newly ringed birds. Pairs of fieldworkers walked through sub-colonies of incubating birds and ringed birds were targeted for re-capture. Most birds typically stand as they are approached and were caught by one person while the other noted their ring numbers. In this way the entire population of incubating birds at both colonies was surveyed. This was repeated a week later to maximise the probability of re-capturing both birds of incubating pairs – Ascension Island birds spend up to seven days away at sea before returning to relieve their partners of incubation duties [24]. The re-capture effort for each breeding season is reported in table 1. Through re-capture of ringed birds during incubation across multiple breeding cycles, we calculated the mean interval between capture dates in two consecutive incubation periods. Their incubation period is 29 days and birds were captured during 25 days of the incubation period excluding the last day when eggs were ‘pipping’ and the first three days when birds were more prone to desertion [32]. Multiple breeding intervals from the same bird were not independent and were not included in statistical comparisons.

**Table 1:** Summary of investment in, and outputs from, re-capturing adult sooty terns on Ascension Island ringed while incubating between 1996 and 2012 inclusive.

		cumulative total of birds:	
month and year	re-capture effort (hrs)	ringed	re-captured
October 1996	3	149	0
June 1998	3	331	0
November 2000	5	881	0
October 2001	3	1084	2
June 2002	34	2464	9
April 2003	19	2864	52
February 2004^a^	64	4864	215
November 2004^a^	32	5364	379
October 2005	18	5750	495
August 2006	56	6150	699
May 2007	53	9050	960
February 2008^a^	76	11050	1356
December 2008^a^	52	13200	1932
October 2009	22	13200	2048
July 2010	3	13250	2072
April 2011	82	15776	2454
January 2012^a^	47	16276	2721
December 2012^a^	166	17426	3355

a Note that the sub-annual breeding cycle results in birds breeding twice in these years.

The breeding cycle of this pan-equatorially distributed species is composed of periods of courtship, incubation, chick-rearing and migration which we compared between birds on Ascension Island and conspecifics on other islands (data from Schreiber et al. [32] and references therein). Courtship was defined as the period between thousands of birds assembling in ‘towers’ off the breeding area [33] following migration and the start of laying. We have observed courting birds mating on Ascension Island and they then return to sea to ‘feed up’. However, while we suspect that pair bonds are maintained between birds while at-sea, other details of courtship, as they do at their other breeding locations, lack an empirical basis. On Ascension Island courtship includes the ‘night clubbing’ phase when birds land at night in discrete areas [24]. The migration period (in days) of birds was calculated as:
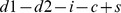
where *d1* and *d2* are the dates of hatching of first chick of the season, and of departure of the last tern the previous season, respectively, *i* is the mean incubation period (i.e. 29 days), *c* is the mean courtship period (i.e. 52 days), and *s* is the mean interval between the first egg date in the two colonies and the date of departure of the last bird from the two colonies.

### Ethics Statement

The research presented here has been approved by the British Trust for Ornithology which is the licencing authority who granted the ringing licence (A4318) to CPW and under which birds were ringed on this UKOT.

## Results

### Population Breeding Interval

The mean interval between first estimated hatching dates in successive breeding seasons was 289.4±10.1 (±95% confidence limits) days (N = 17) at Mars Bay and 288.9±8.7 days (N = 19) at Waterside (figure 1). The mean breeding interval of the Ascension Island population was shorter by 76.1±8.7 days than that of annually breeding conspecifics elsewhere in the species' range (data from Schreiber et al. [32] and references therein). The Ascension Island population has a sub-annual breeding interval of 9.6 months.

### Individual Breeding Interval

**Figure 2:**
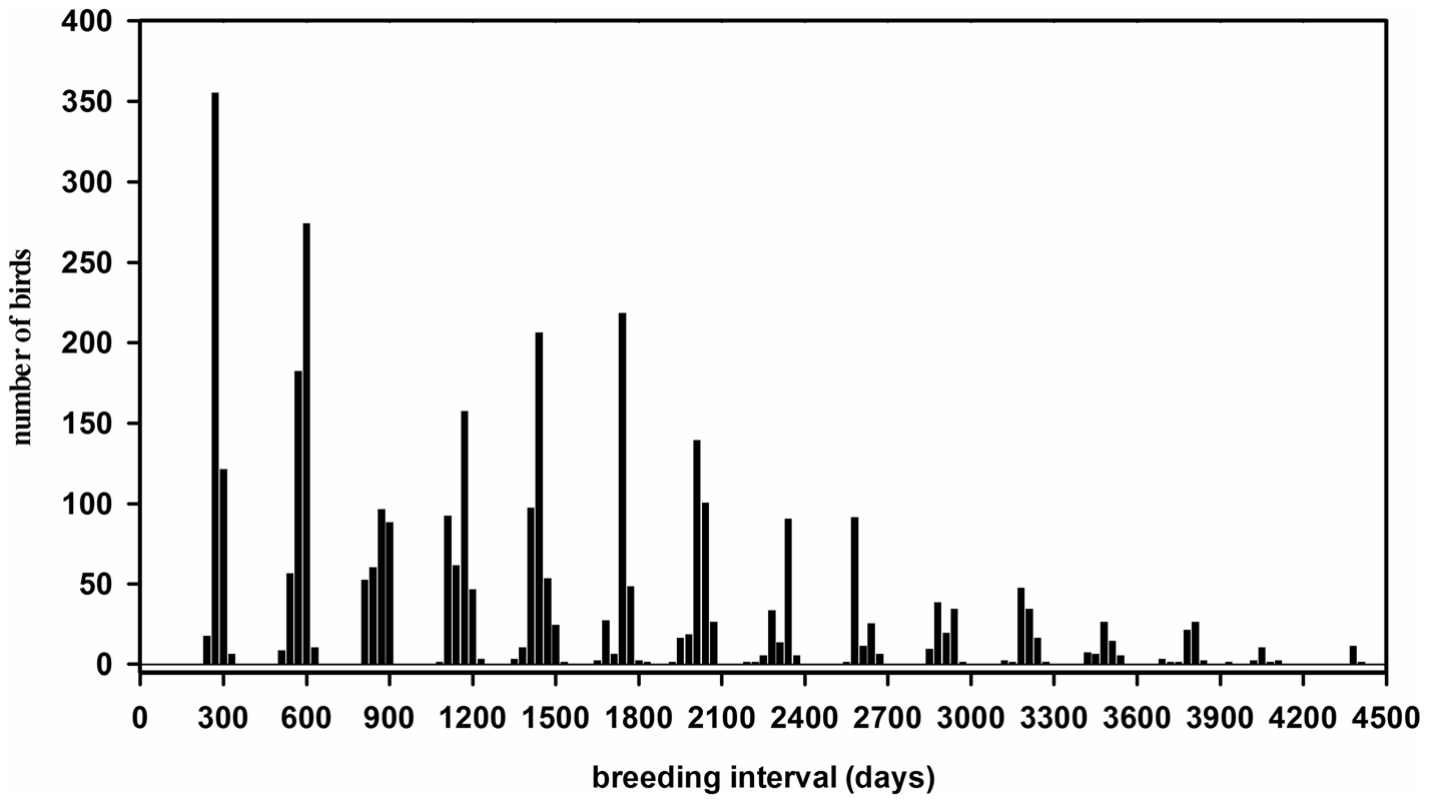
Breeding intervals of individual sooty terns on Ascension Island. The frequency distribution of breeding intervals (bar widths of 30 days) of individual sooty terns based upon their re-capture during incubation following their initial ringing (on day 0) as incubating adults on Ascension Island in the South Atlantic.

Out of a total of 17426 birds that were captured and ringed between 1996 and 2012 (table 1), and over 15 breeding periods, 3353 (or 19.2%) of them were re-captured during incubation during subsequent breeding attempts. Intervals between capture dates showed a well-defined peak at regular intervals of approximately 290 days across many breeding periods (figure 2). The mean interval between dates of incubation for individual birds in two consecutive breeding periods was 290.2±1.4 (±1 standard deviation) days (range: 241–343 days, N = 499) and in three consecutive breeding periods it was 287.6±7.1 days (range: 223–329 days, N = 15). Our results also indicated that some even breed in four successive periods; we captured three incubating birds that showed consistent sub-annual breeding intervals (bird 1: 287, 294 and 243 days; bird 2: 281, 293 and 304 days; bird 3: 279, 267 and 299 days).

### Composition of Sub-Annual and Annual Breeding Cycles

**Figure 3:**
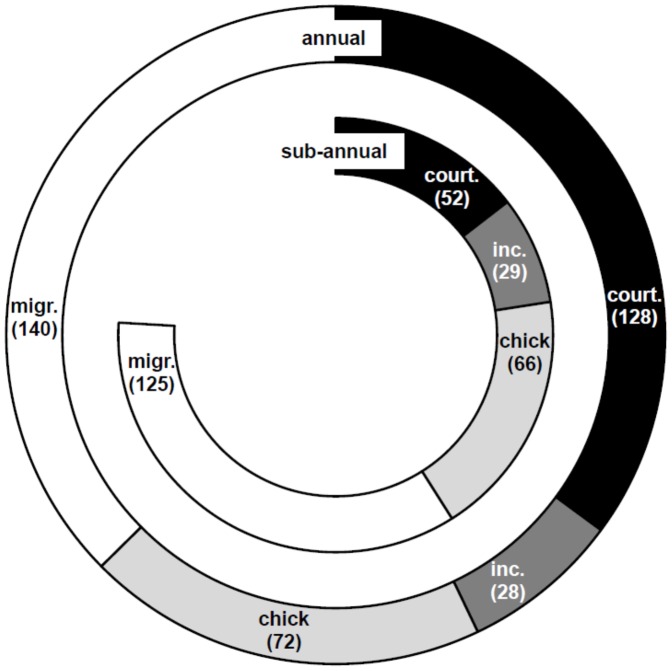
Composition of breeding cycles of sooty terns breeding annually and sub-annually. Relative lengths (in days) of stages (court. – courtship, inc. – incubation, chick – chick-rearing and migr. – migration) in the sub-annual and annual breeding cycles of sooty terns. Data are from this study for sub-annually breeding birds and from Schreiber et al. [32] (and references therein) for annually breeding populations.

A comparison between sub-annually breeding sooty terns on Ascension Island and annually breeding conspecifics elsewhere revealed differences of a single day between incubation periods, six days between chick-rearing periods and 15 days between migration periods (figure 3). However, the mean “courtship” period was 128 days in annual breeders (range: 90–165 days, N = 4) compared with 52 days in sub-annual breeders (range: 38–62 days, N = 3) (figure 3). This difference of 76 days corresponds exactly with the difference between breeding intervals.

## Discussion

We provide strong evidence of consistent sub-annual breeding at both the individual and the population levels of sooty terns breeding on Ascension Island. Birds bred twice in the calendar years 2004, 2008 and 2012 and sub-annual breeding intervals translated into breeding peaks in different months of the year over the course of this 16-year study (figure 1). Ringing and re-capture data indicated that individual birds bred with a sub-annual breeding interval of 9.6 months (figure 2). Finally, we have shown that sub-annual breeders on Ascension Island exhibit a severely truncated courtship phase in their breeding cycle compared with that of annually breeding conspecifics from elsewhere (figure 3). The 76-day truncation of courtship exactly matched the difference between breeding intervals of sub-annually and annually breeding populations of birds.

### Long-term Stability of the Population's Sub-annual Breeding Cycle

The sub-annual breeding interval of the sooty tern population on Ascension Island appears to have remained constant for at least the last 140 years. From historical records (Chapin [20] and references therein) there is qualitative evidence for a breeding interval of between eight and 10 months since the 1870s. The starts of breeding periods were recorded in August 1875, March 1876, early in 1877 and again in November of the same year. The mean breeding interval for the population of 291 days between 1942 and 1958 [33] is nearly identical to the 289 days that we obtained (figure 1). In the 1960s K.E.L. Simmons (unpublished data) recorded first-egg date at Waterside on 29 January 1962, 17 November 1962 and 21 September 1963 representing periods of 292 and 308 days, respectively.

### Individual Sub-annual Breeding and Productivity

Ashmole [24] provided only limited evidence for a sub-annual breeding interval of individual sooty terns breeding on the island. In contrast, we provide robust evidence from 3353 ringed birds that the breeding interval is indeed sub-annual and consistent over 15 breeding seasons (figure 2). We only re-captured three birds in more than three consecutive seasons that had clearly not skipped breeding periods but many more such birds need to be re-captured if we are to conclude that Ascension Island birds do not breed in a period if they are successful in the previous breeding period. We know that egg production per pair on Ascension Island is modally one per sub-annual breeding period but this translates to 1.22 eggs per annum when account is taken of the sub-annual breeding period [29]. Although annually breeding conspecifics elsewhere also produce only one-egg clutches [32], the increased annual egg production of sub-annually breeding Ascension Island birds is mitigated by fewer re-laying attempts. Only 13% of birds on Ascension Island re-lay after a failed breeding attempt [24] while as many as 90% of failed breeders re-lay in the population breeding on The Seychelles [34]. Therefore, reproductive output of Ascension Island birds (i.e. fledged chicks per year) may be similar or even lower than that of conspecifics elsewhere.

### Key Aspects of the Sub-annual Breeding Cycle

Compared with the annually breeding populations of sooty terns, Ascension Island birds appear able to breed sub-annually by reducing the courtship period by 76 days. This matches the difference between annual and sub-annual breeding intervals. There appear to be some fundamental differences in the breeding biology of the population on Ascension Island compared with elsewhere. For example, laying peaks towards the end of the laying period 40–60 days after the first egg is laid on Ascension Island [30]; in annual breeders laying is more synchronised such that of all eggs produced by the population, 85% and 90% were laid in 28 days on Europa Island [35] and Johnston Atoll [36], respectively, 75% were laid in nine days on The Seychelles [34], and 100% were laid in 21 days on the Dry Tortugas [32]. In the 1960s during courtship birds visited the Ascension Island breeding colony each night in relatively low numbers (i.e. approx. 5000 birds out of a total breeding population of 750000 [24]). On Johnston Atoll in the Pacific where birds breed annually, Harrington [36] caught birds during courtship but found only 5% of 756 birds had obvious brood patches suggesting most were not ready to lay. After migration pairs of birds land on Ascension Island to copulate, depart for pelagic feeding grounds for another 2–3 weeks and then return to the island when they immediately lay [24]. Given moult data from different populations of both annually and sub-annually breeding sooty terns [37], all birds appear able to complete courtship, breeding and moult during nine to 10 months. However, while annually breeding birds appear to delay breeding, Ascension Island birds appear to breed with minimal delay once they have attained breeding condition and once they have a quorum (i.e. a minimum number of birds to start breeding as a colony [31]). What role social stimulation plays in such an assemblage becoming quorate and, hence, in the timing of breeding remains largely unresolved [31] and further research should examine whether breeding synchrony between colony members affords fitness benefits to sooty terns as it does for other species (e.g. European herring gulls [*Larus argentatus*] [38]). However, it remains unclear how social stimulation alone might explain the persistence of the sub-annual breeding cycle of Ascension Island birds.

### Sub-annual Breeding in the Absence of Ecological Signals

**Figure 4:**
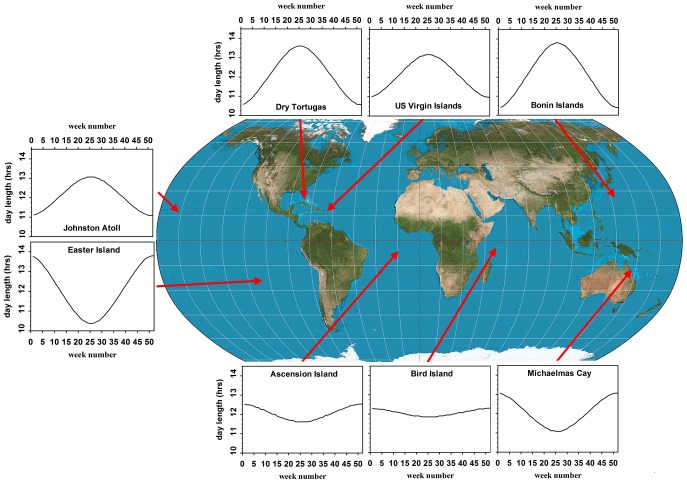
Breeding colonies and day length in sooty tern populations which breed annually and sub-annually. Annual changes in day length during 2012 at colonies where sooty terns breed annually and at Ascension Island where they breed sub-annually.

Breeding seasons evolve because most habitats are exposed to seasonality which results in organisms receiving maximum fitness benefits by restricting their breeding attempts to the most favourable times of the year [3]. Since most habitats are strongly seasonal, most animal species will be annual breeders and consistent sub-annual breeding at the population level should be extremely rare. Our findings of consistent sub-annual breeding at both population and individual levels must reflect a remarkable ecological consistency. In reaching this conclusion, we have dismissed day length as playing any fundamental role in controlling the timing of breeding of sooty terns on Ascension Island despite it having potent and pervasive influence over the timing of breeding and moult in some birds [39]. It is interesting that day length on Ascension Island is relatively constant across the year but is even more so on Bird Island in The Seychelles (figure 4) where the sooty tern population breeds annually [34]. At other major breeding sites within its range (some shown in figure 4), other populations breed annually too [21]. Looking beyond day length, we turn to life history for further explanations. Birds appear to truncate the courtship phase of the breeding cycle which allows them to return to Ascension Island to re-establish pairing and to copulate before they depart once more to ‘feed up’ in preparation for egg laying and incubation. This is similar to the pre-laying exodus of various petrel species [40], but also to sooty terns at the Dry Tortugas in Florida, USA that return briefly to the breeding colony 1–2 months before laying starts [41].

Wingfield [2] examined the structure of the avian annual cycle and argued that at its simplest it could be partitioned into the three distinct life-history stages of breeding, moult and over-wintering. Sooty terns breeding on Ascension Island do not ‘over-winter’ but, instead, their sub-annual cycle occurs at variable times of the calendar year across many breeding attempts. They may have reduced their sub-annual cycle to just two life-history stages (i.e. breeding and moult). If some species of birds (e.g. common starling [*Sturnus vulgaris*]) are kept experimentally under constant equatorial photoperiods and temperature, and with access to *ad libitum* food (i.e. with major sources of environmental variation removed), they show repeated circannual rhythms of gonadal maturation and moult which typically last less than 12 months and are often nearer to nine months [42], [43]. In these cycles, moult tends to start at the time of gonadal regression, and renewed gonadal maturation occurs at the end of moult (i.e. the cycle consists of just the two life-history stages). While birds must breed, they must also moult to maintain plumage quality and it is advantageous not to overlap these life-history stages significantly because both are nutritionally, energetically and temporally demanding (but see [44]). Therefore, the sum of the time taken for these two stages imposes a physiological constraint which determines the minimum length of the breeding cycle. In Ascension Island sooty terns this is less than 12 months allowing breeding to occur sub-annually.

Bird species generally demonstrate a remarkable diversity in moult strategies between different locations, sexes and age classes [45]. Ashmole [24] found that most sooty terns returning to Ascension Island in night swarms before laying had recently completed moult with only a few yet to complete replacement of outer primaries, middle secondaries or outer rectrices. Furthermore, later in the breeding attempt some birds had already started a new cycle of moult and by the time that the colony had dispersed at the end of the breeding season the vast majority of adult birds were in moult. He concluded that for the population as a whole moult and breeding were alternating. At the level of the individual, he found that incubating birds were never in moult and the loss of primaries or rectrices was always observed only in birds that had lost eggs or chicks (and had not re-laid), or that had chicks close to fledging. Sooty terns from elsewhere in the range demonstrate that the species has a remarkable diversity in moult strategy [45]; while Ascension Island birds demonstrate what appears to be a complete moult between successive breeding periods, birds from Phoenix and Line Islands in the central Pacific show discontinuities between primaries and secondaries, indicating that they had not undergone a complete moult between successive breeding periods [37], which has a six month periodicity at the population level. These Pacific birds may demonstrate a number of moult strategies to maximise their breeding potential: some that raise a chick successfully may miss the next breeding period to undergo a complete moult while others whose breeding attempt fails prematurely may try again in the next breeding period after only a partial moult.

### Conclusions

Despite having studied the Ascension Island population of birds since 1990, we still know little about the physiological constraints on birds of this population in relation to the breeding cycle. Certainly, differences between moult strategies of birds breeding on Ascension Island and elsewhere warrant closer consideration but so too do other aspects of their comparative life history. As figure 2 suggests, sooty terns are certainly capable physiologically of breeding sub-annually but on Ascension Island most fail to breed successfully even if we find many on eggs every 9.6 months. They have a relatively low fledging success (0.31 fledglings/pair; BJH unpublished data) compared with elsewhere (e.g. 0.62 in the Dry Tortugas, 0.58 in The Seychelles [32]). If breeding is successful, birds still breed sub-annually albeit with an interval of 342.0±2.8 days (N = 5; BJH unpublished data). Compared with sooty terns breeding on coral atolls in shallow seas, birds on Ascension Island forage over a deep ocean where prey may be more difficult to locate. This may result in birds having significantly longer foraging ranges [24] and incubation shifts of up to seven days compared with one to two days in other parts of the range such as The Seychelles [34] and the Dry Tortugas [41]. The relatively ‘loose’ synchrony of breeding compared with other colonies may allow Ascension Island sub-colonies to exploit ephemeral food sources more successfully [46] but we are only just learning about where birds from Ascension Island forage and how this relates to their breeding success.

We provide strong empirical evidence for regular sub-annual breeding of a free-living avian species. For the first time we employ robust data to demonstrate that this occurs at the level of both the population (hypothesis 1) and the individual (hypothesis 2). We have also shown that the lengths of some constituent phases of the breeding cycle differ between Ascension Island birds and those elsewhere (hypothesis 3), identifying focal points for future research. Our findings increase understanding of the timing of breeding in relation to prevailing ecological conditions. It is clear that sooty terns breeding on Ascension Island may not experience an annual cycle in the availability of ecological resources but this may be overlain by ENSO and other generally unpredictable events. While this lack of an ecological signal translates into average breeding success per breeding attempt being lower for sub-annual breeders, this strategy potentially provides individual birds with more chances to breed during their lifetimes. Therefore, sub-annual breeding must be advantageous to Ascension Island birds despite their success per breeding attempt being lower. Further research is needed to identify other species with enduring and consistent sub-annual breeding cycles and, as result, to advance our understanding of breeding periodicity of vertebrates through the testing of proximate and ultimate causal explanations of the phenomenon.
